# Intranasal Oxytocin Enhances Connectivity in the Neural Circuitry Supporting Social Motivation and Social Perception in Children with Autism

**DOI:** 10.1038/srep35054

**Published:** 2016-11-15

**Authors:** Ilanit Gordon, Allison Jack, Charlotte M. Pretzsch, Brent Vander Wyk, James F. Leckman, Ruth Feldman, Kevin A. Pelphrey

**Affiliations:** 1 Child Study Center, Yale University, New Haven, CT 06520, USA; 2 Department of Psychology, Bar-Ilan University, Ramat-Gan 5290002, Israel; 3 Autism and Neurodevelopmental Disorders Institute, George Washington University, Ashburn, VA 20147, USA.

## Abstract

Oxytocin (OT) has become a focus in investigations of autism spectrum disorder (ASD). The social deficits that characterize ASD may relate to reduced connectivity between brain sites on the mesolimbic reward pathway (nucleus accumbens; amygdala) that receive OT projections and contribute to social motivation, and cortical sites involved in social perception. Using functional magnetic resonance imaging and a randomized, double blind, placebo-controlled crossover design, we show that OT administration in ASD increases activity in brain regions important for perceiving social-emotional information. Further, OT enhances connectivity between nodes of the brain’s reward and socioemotional processing systems, and does so preferentially for social (versus nonsocial) stimuli. This effect is observed both while viewing coherent versus scrambled biological motion, and while listening to happy versus angry voices. Our findings suggest a mechanism by which intranasal OT may bolster social motivation—one that could, in future, be harnessed to augment behavioral treatments for ASD.

The neuropeptide oxytocin (OT) plays a critical role in social processes^1^. Intranasal OT administration in healthy people impacts mentalizing^2^, empathy^3^, trust^4^, biological motion detection^5^, and emotion recognition^6^. Genetic polymorphisms on the oxytocin receptor gene may confer cumulative risk for social impairment^7^. Thus, OT is a candidate system for involvement in disorders that feature core social impairments, such as autism spectrum disorder (ASD). Several single-administration studies indicate that individuals with ASD demonstrate short-term symptom improvement following OT inhalation^8^,^9^,^10^,^11^; however, chronic administration studies are few and show mixed effects. Initial attempts revealed only modest improvements in primary outcome measures^12^,^13^, although more recent studies show enhancement in social responsiveness in children^14^ as well as social reciprocity in adults^15^. Our understanding of the potential for OT to treat ASD is hampered by a lack of sensitive, objective measures that are designed to illuminate mechanisms of change. Despite cautionary calls^16^ regarding the use of OT to treat ASD prior to understanding its developmental effects, there have been few studies of OT’s effects on brain activity in children, creating a translational gap between the push for clinical use of OT and our limited understanding of the developmental and contextual factors that shape its effects on neural function and resulting behavior.

Especially relevant to OT’s action in ASD may be its effects on social motivation. Pro-social aspects of OT’s impact have been previously documented^17^, such as its ability to increase social bonding^18^, trust^4^, and empathy^2^. [Interested readers may find an exhaustive review of studies of the neural effects of intranasal OT prior to 2013 in ref. 19, and a review of studies conducted subsequent to the publication of that manuscript in Table S1.] These effects are thought to arise in part from OT’s interactions with the mesocorticolimbic dopaminergic pathway (see Fig. 1), which encompasses limbic sites (ventral tegmental area, nucleus accumbens [NAcc], and amygdala [AMYG]) and prefrontal cortex (PFC). These interactions can be observed in OT’s impact on functional connectivity between these brain sites^19^. Dopamine is known to shape motivated behavior by influencing the drive to obtain a reward; OT projections to mesolimbic sites may allow OT to modulate dopaminergic activity along this important pathway^1^,^20^, thereby increasing the drive for affiliation. These pro-motivational effects of OT are only one aspect of this hormone’s complex function. OT’s influences on reward pathways are also likely to modulate the saliency of social stimuli^21^. As such, its impact on social motivation may be part of a broader role in increasing social salience^22^,^23^ by regulating attention to crucial social cues, regardless of their valence. According to the Social Salience model^23^, OT’s effects, whether positive or negative, are context dependent. OT’s pro-social effects on social motivation thus may be best observed in the presence of positive (versus negative) social cues, and via targeted assessment of changes in communication along the mesocorticolimbic pathway.

Individuals with ASD may experience dysfunction of the social motivation system. The social motivation hypothesis of ASD argues that reduced social drive leads to inattention to social information and consequent failure of developmental specialization in experience-expectant social brain systems, including action and voice perception systems^24^. Supporting this theory is evidence that individuals with ASD show less activity in mesolimbic sites (i.e., NAcc, AMYG) in response to social rewards than do typically developing controls^25^,^26^. Moreover, a recent chronic administration study in adults with ASD revealed that the behavioral improvement induced by OT was related to enhancement of resting-state functional connectivity between mesocortical sites (i.e., anterior cingulate cortex and dorso-medial PFC^15^). These lines of evidence led us to hypothesize that diminished social motivation in ASD might stem from reduced mesocorticolimbic connectivity, and that OT administration could increase this connectivity, addressing the theoretical crux of social dysfunction in ASD according to the social motivation hypothesis. Specifically, we predicted that intranasal OT would enhance effective connectivity between subcortical (NAcc and AMYG) and cortical (PFC) sites on the mesocorticolimbic reward pathway, as well as between mesolimbic sites and cortical association regions involved in processing socially meaningful perceptual information.

To test this hypothesis, we employed a randomized, double-blind, placebo-controlled crossover design in which 21 children with ASD (8–16.5y, 18 boys; see Table 1 for demographic and phenotypic characteristics) received either OT or placebo (PLC) prior to an initial fMRI scan. In the subsequent visit, children blindly received the other agent prior to a second fMRI scan. One participant could not complete both visits owing to a scheduling conflict and was excluded. Given the developmental nature of ASD and the opportunity to inform early treatment approaches, we chose to focus on children and adolescents with ASD. We did not study a comparison group because, as determined by our Human Investigations Review Committee, the scientific benefits of studying typically developing (TD) children did not compare favorably with the greater than minimal risks of a study involving the administration of OT to children. Further, a within-subjects design focused on children with ASD adequately addressed our research aims. With this single-challenge study, we aimed to evaluate, not lasting therapeutic benefit, but rather target engagement within the clinical population of interest. Here, we use noninvasive imaging to demonstrate ‘proof of biology’^27^, providing evidence that intranasal OT engages a system (the mesocorticolimbic pathway) that, based on the social motivation hypothesis^28^, we would need to upregulate in order to see therapeutic benefit.

Both fMRI scans included two social perception tasks: a Biological Motion task^29^ that contrasted viewing of a social versus a non-social stimulus (coherent [BIO] versus scrambled [SCRAM] human motion); and an Affective Voices task that contrasted listening to a rewarding versus aversive social stimulus (happy [HAP] versus angry [ANG] voices). We employed passive paradigms in order to assess OT’s effects on social perception in the absence of potentially confounding task demands (e.g., refs 29, 30, 31, 32). We conducted whole-brain main effects analyses to identify brain regions that 1) displayed greater activity to BIO than SCRAM, under OT versus PLC, and 2) either greater or lesser activity during ANG versus HAP, under OT versus PLC. After quality assurance review to exclude datasets contaminated by excessive motion, our final Biological Motion sample included 14 children (11 boys), and our Affective Voices sample included 16 children (13 boys). See Table 2 for data quality characteristics of the full sample and experimental subsamples.

To address our core hypothesis, we conducted psychophysiological interaction (PPI) analyses of fMRI data from these tasks. PPI analysis is a means of identifying regions across the brain whose activity is more highly correlated with that of a seed region in one experimental condition than another; typically, the results are interpreted as reflecting task-based changes in effective connectivity, although connectivity is not directly assessed. Based on our prediction that OT should upregulate communication along the mesocorticolimbic pathway, as well as between this pathway and socially-relevant cortical association regions, we evaluated oxytocin-induced, task-specific changes in effective connectivity with mesolimbic seed regions. Specifically, for Biological Motion, we assessed which regions of the whole brain increased connectivity with NAcc preferentially during BIO versus SCRAM, under OT versus PLC. For Affective Voices, we assessed OT > PLC increases in effective connectivity with NAcc for both the HAP > ANG and ANG > HAP; we also assessed these increases in connectivity with AMYG, given the emotional salience of the affective voice stimuli. Importantly, the model specification for this analysis ensures that results will only indicate effects over and above those explained by either co-activation related to the task main effect or anatomical connections that lead to pervasively correlated activity regardless of task condition^33^.

Finally, in exploratory analyses, we evaluated whether children’s scores on two parent-report measures of social functioning, the Social Responsiveness Scale^34^ (SRS) and the Autism Diagnostic Interview-Revised^35^, were associated with OT-induced connectivity changes. We added the SRS total *t*-score (which assess current ASD symptomaticity) and the ADI-R diagnostic algorithm score A, “Qualitative Abnormalities in Reciprocal Social Interaction” (which assesses historical/most severe symptomaticity) as regressors of interest to the group-level models. This allowed us to determine whether and how OT response profiles might differ based on symptom severity in the social communication domain.

## Results

### Main effects of OT

#### Biological Motion

**Figure 2:**
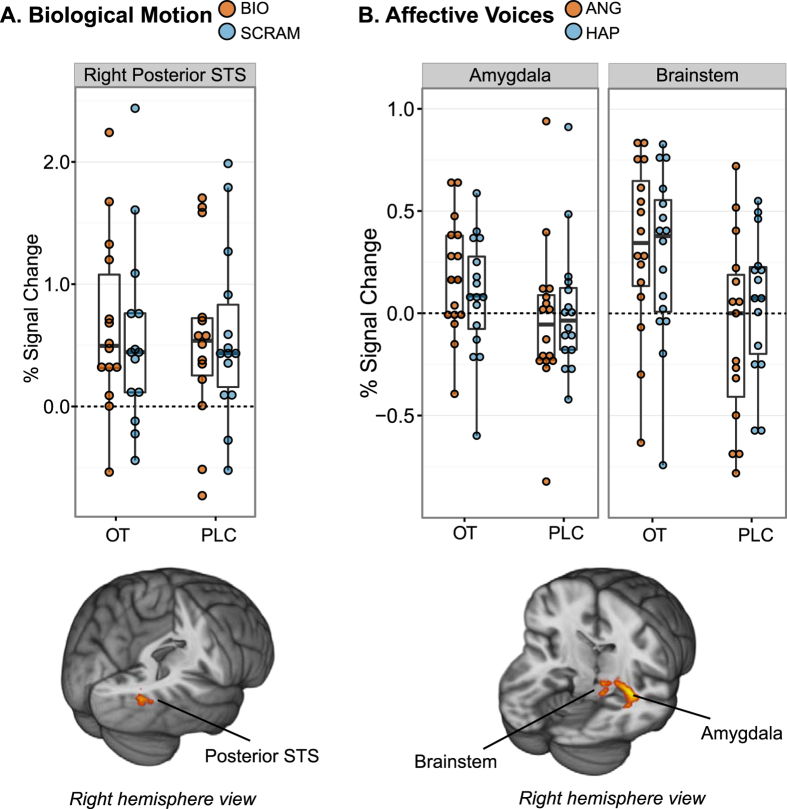
Brain regions in which activation to the experimental contrast of interest was significantly greater under oxytocin (OT) than placebo (PLC). Main effects of OT administration on brain response to (**A**) coherent (BIO) versus scrambled (SCRAM) biological motion and (**B**) angry (ANG) versus happy (HAP) voices are depicted. Results are displayed on the MNI standard brain template, cluster-corrected at *p* < 0.05 and *Z* = 2.3. Bar graphs depict group mean percent signal change to each experimental condition, with standard errors.

Right posterior superior temporal sulcus (pSTS) demonstrated a more strongly positive response to BIO than SCRAM under OT versus PLC; under PLC, brain response to BIO versus SCRAM stimuli was not strongly differentiated at this site (Fig. 2A, Table 3). A number of regions also showed attenuated deactivation under OT versus PLC; see Table S2. BIO > SCRAM task-based effects under OT alone and PLC alone are reported in the Supplementary Results, Fig. S1, and Table S3.

#### Affective voices

Regions of right brainstem and right AMYG demonstrated greater activation to ANG than HAP, specifically under OT versus PLC; under PLC, these sites demonstrated deactivation to ANG voices, compared to relatively less deactivation or minimal activation to HAP (Fig. 2B, Table 3). A number of additional sites demonstrated patterns of attenuated deactivation under OT versus PLC; see Table S2. Whole-brain analysis detected no clusters for which HAP was greater than ANG for OT > PLC. ANG > HAP and HAP > ANG task-based effects under OT alone and PLC alone are reported in the Supplementary Results, Figs S2 and S3, and Table S4. We note also that conjunction inference^36^ identified regions of bilateral superior temporal gyrus that demonstrated an OT > PLC effect regardless of emotional valence (i.e., ANG > fixation ∩ HAP > fixation). See Fig. S4 and Table S5.

### OT effects on connectivity

#### Biological Motion

**Figure 3:**
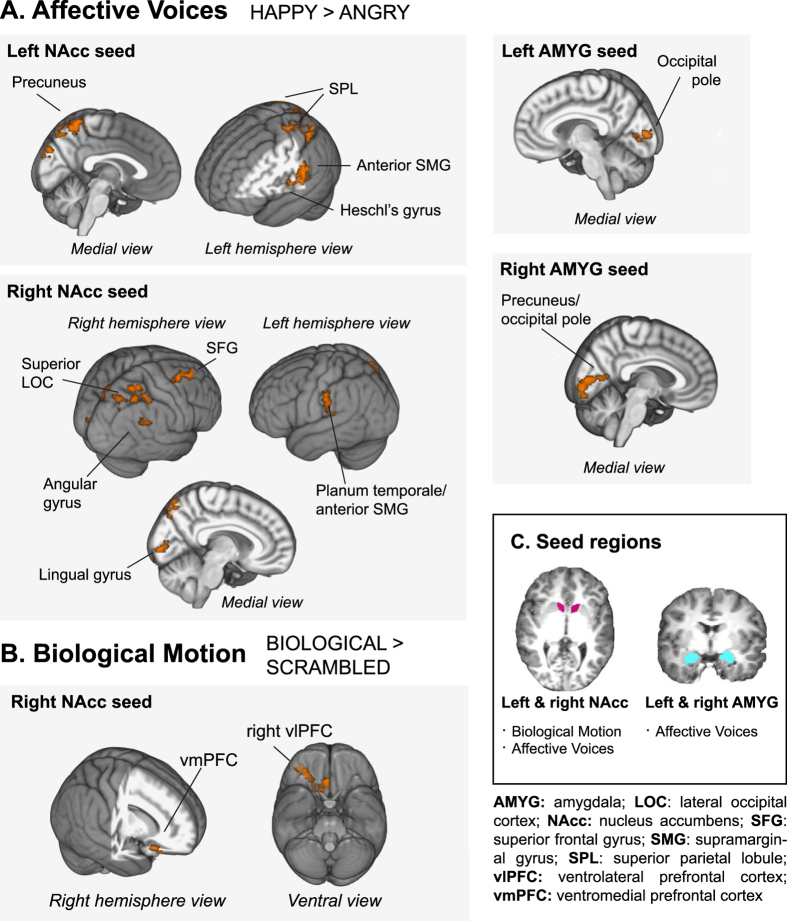
Oxytocin (OT)-induced increases in effective connectivity as assessed via whole-brain psychophysiological interaction (PPI) analysis. Results of PPI analysis, indicating regions with significantly greater connectivity to the seed of interest for (**A**) happy greater than angry voices and (**B**) coherent versus scrambled biological motion, under OT versus placebo (PLC), are displayed on the MNI standard brain template, cluster-corrected at *p *< 0.05 and *Z* = 2.3. (**C**) Segmentation of seed regions from one representative participant’s T1-weighted anatomical image.

PPI analysis was used to identify regions whose activity was more correlated with that of NAcc while viewing BIO compared to SCRAM, under OT versus PLC. OT administration was associated with a greater PPI between right NAcc and a region of ventral PFC. This cluster had a peak in right ventrolateral PFC (vlPFC) and extended into right ventromedial PFC (vmPFC). There were no clusters associated with the left NAcc seed. See Fig. 3B and Table 4.

#### Affective Voices

PPI analysis localized regions whose activity was more correlated with the seed of interest either for HAP > ANG or ANG > HAP, under OT versus PLC. OT administration induced connectivity increases with all seeds of interest (left and right NAcc and left and right AMYG) for HAP > ANG (see Fig. 3A). For left NAcc, clusters emerged with peaks in precuneus (PCu), cuneus (Cu), left anterior supramarginal gyrus/Heschl’s gyrus (aSMG/HG), and right temporo-occipital fusiform gyrus (FFG). For right NAcc, the pattern of results was similar, with significant clusters arising in PCu, right middle frontal gyrus, left planum temporale/aSMG, intracalcarine cortex, and right angular gyrus. For both the left and right AMYG seeds, clusters emerged in posterior occipital regions. For left AMYG, the peak of this cluster was localized to the occipital pole, and for right AMYG, this cluster peaked in PCu. No significant findings emerged for the ANG > HAP contrast. Table 4 characterizes these cluster peaks; local maxima can be found in Table S6.

### Relationship between symptom severity and OT-induced changes in connectivity

**Figure 4:**
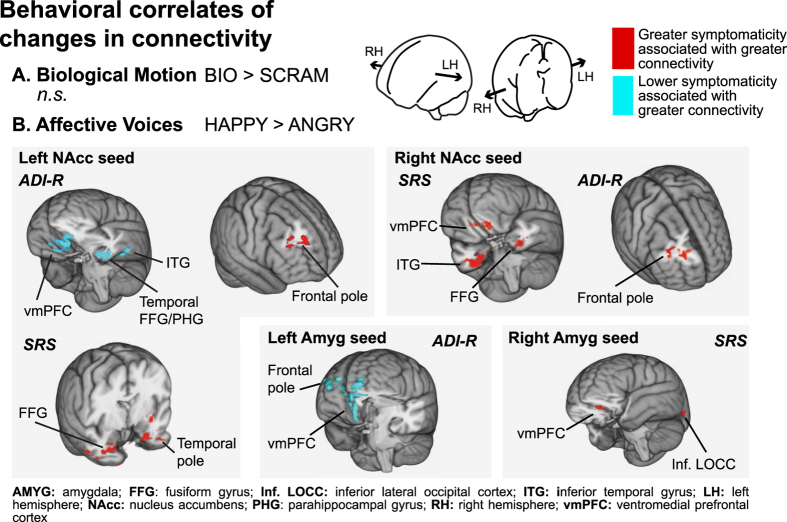
Brain regions for which oxytocin (OT)-induced connectivity changes (OT > placebo) for Happy versus Angry voices were associated with levels of social functioning, as assessed via the Social Responsiveness Scale (SRS) total *t*-score and *t*he Autism Diagnostic Interview-Revised (ADI-R) diagnostic algorithm A: “Qualitative Abnormalities in Reciprocal Social Interaction”. Higher scores on the ADI-R or SRS indicate greater symptom severity. Results are depicted in MNI space, with regions displaying negative correlations (lower symptomaticity associated with a higher PPI value) in cyan and regions displaying positive correlations (greater symptomaticity associated with a higher PPI value) in red. Brain outline images at top were adapted in part from an illustration by Patrick Lynch, medical illustrator, and C. Carl Jaffe, MD, cardiologist (available at https://commons.wikimedia.org/wiki/File:Cranial_nerve_VII.svg), licensed under CC BY 2.5, 2006.

During Biological Motion, we found no brain regions for which a significant association between symptom severity and OT-induced change in BIO > SCRAM effective connectivity existed. However, during Affective Voices, a higher SRS score (indicating greater impairment) was associated with greater HAP > ANG PPI values between seeds of interest and a variety of brain regions. Specifically, for the seed in left NAcc, significant clusters emerged in left temporal pole and right FFG; for right NAcc, clusters emerged in vmPFC, left FFG, and right anterior inferior temporal gyrus (ITG); and for right AMYG, clusters were observed in vmPFC and left inferior lateral occipital cortex (see Fig. 4 and Table S7). Consistent with the SRS findings, a higher ADI-R social subscale score (indicating greater impairment) was associated with greater HAP > ANG PPI values between left AMYG and right frontal pole as well as between either left or right NAcc and vmPFC. (See Fig. 4 and Table S8.)

Conversely, a number of brain regions demonstrated a negative correlation between ADI-R score and OT-induced change in HAP > ANG PPI value, such that greater OT-induced increases in connectivity in these brain regions were associated with a history of less severe social symptomaticity. Specifically, for the left NAcc seed, regions of vmPFC, left ITG, and left FFG displayed this pattern; and for the left AMYG seed, right frontal pole and vmPFC displayed this pattern. (See Fig. 4 and Table S8).

## Discussion

OT administration to children and adolescents with ASD was associated with increased activity in brain regions important for social-emotional perception. In the visual domain, consistent with previous research^29^,^37^, OT enhanced response to human motion versus control stimuli in right posterior superior temporal sulcus (pSTS), a brain region that has been consistently characterized as playing a key role in biological motion perception in neurotypical individuals^38^,^39^. Further, we have previously observed that in contrast to TD children, children with ASD who view this paradigm tend to show lower human-motion-selective activity in pSTS^29^. The OT-induced upregulation of right pSTS response to human motion in our sample of youth with ASD suggests that OT administration may have the potential to promote a more neurotypical profile of engagement during a common social perception task.

In the auditory domain, OT enhanced activation to ANG versus HAP voices in right brainstem and in a region with a peak in right hippocampus but the majority of its extent contained within right AMYG. The AMYG is a region which has been implicated in OT’s effects in TD individuals^40^,^41^ as well as adults with ASD^42^. Similarly, OT has been experimentally demonstrated to facilitate hippocampal activity via processes that improve the signal-to-noise ratio^43^. The upregulation of these regions (which are key to the encoding of emotionally salient stimuli^44^) when perceiving angry voices suggests a particular need for caution in the clinical use of OT. While our effective connectivity analyses (discussed below) suggest that youth with ASD, when exposed to positive social stimuli under OT, show upregulation of reward systems, these findings suggest that OT may also upregulate encoding of *negative* social experiences when these occur. This would be consistent with findings in neurotypical samples indicating that, in addition to its prosocial effects, intranasal OT can also upregulate adaptive but potentially undesirable behaviors like envy and gloating^45^ and defensive aggression toward outgroup members^46^. Thus, when administering OT, it may be particularly important to manage the patient’s social-emotional environment, to ensure that the experience is therapeutic, rather than iatrogenic and potentially socially de-motivating.

Across visual and auditory modalities, OT increased effective connectivity both within the mesocorticolimbic reward pathway and also between mesolimbic sites and cortical regions that contribute to social perception. These effects were specific to exposure to social versus nonsocial information (Biological Motion), and to socially rewarding versus socially aversive stimuli (Affective Voices). These results support our hypothesis that OT increases communication among neural systems relevant to social motivation in ASD. In the visual domain, inhalation of OT (relative to PLC) increased connectivity within the mesocorticolimbic pathway during the perception of coherent biological motion, specifically between NAcc and frontal regions (i.e., vm/vlPFC) known to be involved in representing reward value, social meaning, and social intentions^47^,^48^. In the auditory domain, OT increased connectivity between NAcc and cortical regions involved in auditory/voice processing and social analysis (e.g., auditory cortex [aSMG/HG] and PCu); this increase was selective to the perception of happy, but not angry, voices. Effective connectivity between AMYG and PCu (known to be involved in social cognition and communication^49^), as well as the occipital poles, was enhanced during the perception of happy voices.

During biological motion perception, we found that OT increases connectivity between NAcc and prefrontal regions, including vmPFC and orbitofrontal cortex (OFC). Each of these sites plays a role in motivated behavior. Animal work has shown that variations in OT expression or receptor density in striatal regions, including NAcc, underlie individual- and/or species-level differences in partner preference, monogamy and maternal behaviors^50^,^51^,^52^. OT receptor density in NAcc is linked to variability in responding to pups among nulliparous female prairie voles, with higher densities related to maternal behaviors, and lower densities related to ignoring or attacking pups^53^. In human mothers, NAcc is more active when watching their own versus an unknown infant; moreover, synchronous mothering behavior is related to higher circulating OT levels as well as greater activations in NAcc^54^. This suggests that OT signal in NAcc may increase the appetitive value of a social target and its associated cues, tending the organism towards affiliation. Extensive work indicates that vmPFC and nearby OFC support reward value coding^48^; through interactions with NAcc, these regions may help value signals be translated into action^55^. OT increases OFC activity in response to interpersonal touch in healthy adults, as well as the perceived pleasantness of this touch—effects inversely related to subclinical autistic traits^56^. Previously, greater effective connectivity between NAcc and vmPFC has been linked to a higher individual-specific motivational value for a particular stimulus^57^. Thus, increased NAcc-vmPFC/OFC connectivity during biological motion perception may indicate increased valuation of the social stimulus under OT versus PLC.

While participants listened to emotional voices, intranasal OT increased the connectivity between NAcc and primary auditory cortex, specifically to happy versus angry voices. Consistent with the results from the biological motion paradigm, this finding may also indicate increased value for a socially rewarding stimulus—here, for happy voices. NAcc connectivity has previously been linked to the reward value of auditory stimuli in the domain of music. For healthy individuals, listening to pleasant music is associated with increased connectivity among mesolimbic regions^58^, and placing a greater value on a musical stimulus is associated with an increase in connectivity between NAcc and auditory cortices^57^. During happy voice perception, OT also induced increases in connectivity with PCu from both mesolimbic sites, with NAcc interacting with more anterior regions of PCu and AMYG interacting with more posterior PCu regions. Anterior PCu, has been linked to self-referential processing^59^, and OT increases activity in this region during interpersonal touch^56^. Increased NAcc-PCu connectivity might indicate that happy vocal cues gain relevance to the self and that this increased self-relevance figures into calculations of motivational value. AMYG-posterior PCu interactions have different implications. The AMYG has multiple, complex roles in emotional experience, motivation, and learning. It has been suggested that AMYG helps the individual detect salient stimuli (particularly socio-emotionally salient stimuli), heightens arousal in response to these stimuli, and facilitates learning and remembering their reward value^20^. Posterior PCu has been implicated in episodic memory retrieval^59^. Together, these findings suggest that OT-induced increases in AMYG-posterior PCu interactions might be related to remembering previous experience with happy cues and their reward value. We note also an unexpected finding of OT-induced upregulation of effective connectivity between AMYG and primary visual regions (e.g. occipital pole) for the HAP > ANG contrast. We note that while this pattern seems unusual in a task with auditory stimuli, previous work has established that crossmodal activation (specifically, activity in primary visual areas in response to auditory stimuli) can be observed in neurotypical individuals^60^,^61^; this effect has even been documented in relation specifically to selective attention to emotional versus neutral voice stimuli^62^. Investigators have speculated that this phenomenon may in some cases be related to a cross-modal priming effect^63^ or to access of visual imagery^62^,^64^,^65^; we feel this is the explanation that may best suit our data. However, given the post-hoc nature of this interpretation, further investigation is required to validate the finding.

Our findings indicate that by upregulating communication among regions related to motivational valuation, social perception, thinking about the self, and reward memory, OT may facilitate social motivation. In individuals with ASD, such an effect may be clinically meaningful; many of the processes we influenced via OT administration are dysfunctional in ASD, where the social world may have reduced reward value^25^,^26^, and neural specialization for social perception is decreased^29^. We note that the effects we observed were context-specific; OT effects on systems underpinning social motivation are best observed in the context of rewarding social stimuli. One reason for the mixed clinical effects of chronic OT administration observed thus far^12^,^13^ may be lack of sufficient control over the experiences to which individuals with ASD are exposed during OT’s window of effect. The selective effects on connectivity demonstrated here suggest that, to target social motivation, OT would be best administered in tandem with thoughtfully designed experiences of rewarding social stimuli and interactions.

We identified a number of sites for which OT-induced connectivity changes during HAP > ANG were associated with the severity of social deficits. Children whose parents endorsed more severe current symptomaticity (via SRS) demonstrated greater increases in connectivity between bilateral NAcc or right AMYG and 1) temporo-occipital sites relevant to social perception, and 2) frontal sites (e.g., vmPFC) relevant to social motivation. Children with a more severe symptom history (as assessed via ADI-R) also showed greater increases in connectivity between right NAcc and frontal pole. A less severe symptom history was associated with greater increases in connectivity between left NAcc or left AMYG and vmPFC. We note that we observe an apparently contradictory pattern: a negative relationship between ADI-R-assessed social symptomaticity and vmPFC connectivity, and a positive relationship between SRS-assessed social symptomaticity and vmPFC connectivity. In our affective voices subsample, ADI-R and SRS scores were not significantly correlated with each other (*r*(14) = 0.28, *p* = 0.300), presumably due to differences in the children’s historical and current social symptom presentation. While speculative, we suggest that the seemingly contradictory results displayed in Fig. 4 are related to differences in the predictive power of historical social impairment versus current symptomaticity. Other studies in clinical populations also show that OT’s effects may vary depending on the nature and severity of an individual’s symptoms^66^. These findings illuminate another aspect of contextual specificity of OT’s effects; not just environmental, but also *person*-specific factors are important to predicting how OT will operate. We propose that OT administration can lead to beneficial effects on neural function in a range of individuals with ASD, but that understanding individual symptomaticity is necessary to predict variability in OT’s therapeutic potential.

A potential limitation of the present work is the inclusion of participants who were on a medication regimen in the sample. While ideally only unmedicated participants would be utilized in an OT administration study in order to preclude potential interactions with other medications, realistically, a not insignificant proportion of children with ASD are on medications to control comorbid symptoms^67^,^68^. Moreover, families who are using psychiatric medication already may be more likely to be interested in participating in research that involves administration of a pharmacological agent. Thus, we were constrained to accept subjects who were on a preexisting medication regimen. Our within-subjects design is intended to help control for this and other factors that may have introduced noise into our sample.

Another limitation of the current report is the small number of participants and specifically the relatively small number of girls in our sample. We note, however, that our sample is one of the only samples to include children with ASD as young as 8 years old and that the distribution of boys and girls accurately reflects the skewed sex ratio of ASD diagnosis and is, to our knowledge, the first OT administration study in a pediatric population to include any girls whatsoever^69^. Given the sexually-dimorphic effects of OT^1^, future work should address this gap in the literature. Future work should also address questions regarding dose effects of a single OT administration, and compare OT’s effects on children with ASD to its effects in TD children.

Our results demonstrate that, in accordance with the assertions of the Social Salience^23^ hypothesis, OT administration has differential effects depending on the social cues present. Thus, during the Affective Voices task, we observe an increase in AMYG and brainstem activity to angry (versus happy) voices under OT. However, we also find that during happy (versus angry) voice perception, OT upregulates effective connectivity along a major reward pathway. In a naturalistic social situation, it would be unusual for an individual to process both happy and angry stimuli at virtually the same time. Thus our findings support the notion that, in the normal course of social interaction, OT serves different functions in different contexts^22^; when highly salient (but not rewarding) angry cues are present, it upregulates AMYG response, whereas when happy cues are present, OT facilitates communication among sites that are important for social motivation.

In summary, employing an experimental therapeutics approach, we evaluated effective connectivity between mesocorticolimbic reward circuits and social perception regions as a potential target for intranasal OT in ASD. OT enhanced effective connectivity between mesolimbic sites and cortical regions involved in value coding and social perception in children and adolescents with ASD. Together with recent findings showing that OT can increase processing efficiency at the neuronal level by optimizing signal-to-noise during cell firing^43^, and our own neuroimaging work demonstrating OT-enhanced neural attunement to visual social cues^37^, these results show that in ASD, OT may not only serve to enhance neural activity to “preferred” stimuli, but also improve communication among brain regions involved in social motivation and perception. While we have recently reported on OT’s ability to enhance the function of several key nodes of the social brain in ASD^37^, the current results illustrate a discrete aspect of OT’s effect captured by measures of connectivity, highlighting the importance of assessing change not only in localized brain regions but also at the neural systems level. By impacting mesocorticolimbic connectivity, OT may create a neural background in individuals with ASD that could bolster the motivation to interact socially and allow them to be more open to learn social engagement skills during treatment. These findings lead us to believe that intranasal OT, via its effects on the neural underpinnings of social motivation, could improve response to behavioral interventions that provide a rich array of social learning opportunities.

## Methods

### Participants

21 8–16.5y children (18 boys) were enrolled, with ASD diagnosis confirmed using the Autism Diagnostic Observation Schedule^70^ and the Autism Diagnostic Interview – Revised (ADI-R)^35^. The Yale University Human Investigations Committee approved this study. All methods were carried out in accordance with the approved guidelines. Each participant’s parent(s) provided written informed consent and children provided written or verbal assent. One participant could not complete both visits and was excluded. Due to motion artifact, the complete datasets from 6 children were excluded from analyses of the Biological Motion task, and 4 complete datasets were excluded from the Affective Voices task. (See “FMRI Data Analysis: Quality Assurance”). Our final Biological Motion sample included 14 children (11 boys), and our Affective Voices sample included 16 children (13 boys). Demographics and quality metrics of the analyzed samples are provided in Tables 1 & 2. A more detailed breakdown of the types of medications in use by our participants can be found in Table S9.

### Drug Protocol

The drug protocol has been described previously^37^. We used a randomized, double blind, placebo-controlled crossover design. The study consisted of two visits (one OT, one PLC) separated by a minimum of 72 hours (average: 21 d, range: 3–78 d). Intranasal doses of 60 international units of OT (IU)/mL, were prepared by the research pharmacy at Yale New Haven Hospital using OT, United States Pharmacopeia (Medisca). Doses were prescribed according to participant age. Older participants (aged 16–19 y) received a dose of 24 IU (two puffs per nostril), in accordance with most studies of intranasal OT in adults^71^. Twelve- to 15-y-olds received 75% of the adult dose or 18 IU (three puffs overall). The youngest age group (aged 7–11 y) received 50% of the typical adult dose (12 IU or one puff per nostril). PLC and OT spray containers were prepared to look identical and were counterbalanced to be randomly assigned by the pharmacy as well. Researchers, as well as participants, were blinded to the content of the spray. We used age-dependent dosing (as suggested by the scant OT inhalation studies in children^69^), with age as a proxy for size/weight. A more extensive description of the OT administration protocol can be found in ref. 37.

### Imaging Parameters

Images were collected at the Yale University Magnetic Resonance Research Center on a Siemens 3T Tim Trio scanner equipped with a 32-channel head-coil. Whole-brain T1-weighted anatomical images were acquired using a Siemens sagittal integrated parallel imaging sequence (TR = 2000 ms; TE = 2.96 ms; flip angle = 7°; FOV = 256 mm; image matrix 256 mm^2^; voxel size = 1 mm^3^; 160 slices; NEX = 1). Whole-brain functional images were acquired using a single-shot, gradient-recalled echo planar pulse sequence (TR = 2000 ms; TE = 25 ms; flip angle = 60°; FOV = 220 mm; image matrix = 64 mm^2^; voxel size = 3.4 mm × 3.4 mm × 4 mm; 34 slices) sensitive to blood oxygen level dependent (BOLD) contrast. Functional slice prescription was parallel to the AC-PC line and covered the entire cerebrum and top half of cerebellum. For the biological motion perception paradigm, 164 volumes were acquired, the first ten of which were discarded to allow for magnetic saturation effects. For the affective voices paradigm, 100 volumes were acquired, the first five of which were discarded.

### Experimental Paradigms

Herein, we describe effects observed during two experimental paradigms conducted following OT administration: a biological motion perception paradigm and a vocal affect perception paradigm. Children also participated in an emotion recognition paradigm, reported elsewhere^37^. Each of these tasks (emotion recognition, affective voices and biological motion) were presented to participants consecutively in the MRI scanner starting at the beginning of the functional scan. Functional scanning began 45 minutes following OT/placebo administration as per the gold standard in OT nasal administration studies. During Biological Motion, participants viewed blocks (6 per condition, 24 s each) of coherent point-light displays of biological motion (BIO) interleaved with scrambled (SCRAM) versions of these displays^29^. During Affective Voices, participants listened, eyes shut, to alternating blocks of angry (ANG) and happy (HAP) non-word vocalizations (3 per condition, 15 s each), with listening blocks separated by 15 s silent periods, during which eyes remained shut. Stimuli were taken from the Montreal Affective Voices dataset^30^. Each block contained five utterances (527–1742 ms) separated by brief silences (1258–2473 ms).

### FMRI Data Analysis

#### Quality assurance

For each experimental paradigm, prior to data analysis, we elected to exclude participants where for either visit, the participant had one or more of the following: movement in any dimension ≥4 mm, and/or ≥25% of volumes identified as outliers according to the DVARS metric^72^ such that only participants who displayed acceptable amounts of motion under both the OT and PLC visits were included in analysis. Preprocessing and quality assurance measures were conducted on all datasets. Excessive motion contaminated six datasets from the biological motion paradigm and four datasets from the vocal affect paradigm, leading to the exclusion of these datasets. Table 1 provides an overview of the demographic characteristics of the overall sample and analyzed subsamples. For the biological motion paradigm, 14 out of 20 participants were retained under these criteria for inclusion in final analysis; for the vocal affect paradigm, 16 participants were retained. Motion and signal-to-noise metrics for included participants are listed in Table 2. For the vocal affect perception paradigm, fewer motion outlier volumes were detected for OT than PLC scans (3.6 versus 5.5% of all time points identified as outliers, respectively), although there were no significant differences across treatment conditions as characterized by RMS movement metrics. To determine whether the difference in motion outlier frequency had an effect on our results, we reran all group-level vocal affect analyses with an additional regressor. For each individual, outlier percentage in the PLC scan was subtracted from the outlier percentage in the OT scan to create a difference score, which was entered as a group-mean-centered regressor. The addition of this regressor did not change the results of the PPI analysis.

#### Preprocessing

FMRI data processing was carried out using FEAT (FMRI Expert Analysis Tool) Version 6.00, part of FSL (FMRIB’s Software Library, www.fmrib.ox.ac.uk/fsl). The following pre-statistics processing was applied: motion correction using MCFLIRT^73^; slice-timing correction using Fourier-space time-series phase-shifting; non-brain removal using BET^74^; spatial smoothing using a Gaussian kernel of FWHM 5 mm; grand-mean intensity normalization of the entire 4D dataset by a single multiplicative factor; high pass temporal filtering (Gaussian-weighted least-squares straight line fitting, with sigma = 50.0 s). Registration to high-resolution structural and standard space images was carried out using FLIRT^73^ and then further refined using FNIRT nonlinear registration.

#### PPI seed creation

Seeds for use in PPI analyses were anatomically defined from participants’ T1-weighted images using FSL’s FIRST^75^, a tool for the automatic segmentation of subcortical structures. Initial registration/segmentation was visually inspected to ensure that structures of interest were accurately segmented. These images were then registered to individual functional space using FLIRT, and checked to ensure that no voxels encompassed non-brain material. For the biological motion paradigm, seeds were created in left and right NAcc. For the affective voices paradigm, seeds were created in NAcc as well as bilateral AMYG.

#### Individual level (fixed effects) analysis

First level analysis for both PPI and main effects was carried out in FEAT, with time-series statistical analysis carried out using FILM with local autocorrelation correction^76^. FSL’s “fsl_motion_outliers” tool was used on non-motion-corrected functional data to detect time points corrupted by large motion using the DVARS metric^72^. A confound matrix was generated identifying time points for which the root mean squared (RMS) intensity difference from volume N to volume N + 1 was greater than the 75^th^ percentile plus 1.5 times the interquartile range. This matrix was used to regress out corrupt time points at first level. Standard motion parameters (six regressors representing translations and rotations in the x, y, and z dimensions) were also included as nuisance regressors in the first level model. For main effects analysis, experimental conditions were modeled using a gamma hemodynamic response function (HRF) with temporal filtering applied and a temporal derivative added. For the biological motion perception paradigm, the contrast of interest was BIO > SCRAM; for affective voices, the contrasts of interest were HAP > ANG and ANG > HAP. Second-level fixed effects analyses were used to create within-subjects estimates of these contrasts of interest for OT versus PLC scans.

For PPI analysis, models were specified according to the methods described by O’Reilly and colleagues^33^. The psychological regressor of interest was convolved with a double-gamma HRF with temporal filtering applied and a temporal derivative added. For the biological motion perception paradigm, the psychological regressor was created from the contrast BIO > SCRAM; for affective voices, psychological regressors were created from the contrasts HAP > ANG and ANG >HAP. The physiological regressor was the mean time series from the seed in functional space. The PPI regressor was the interaction term between the psychological and physiological regressors, with the psychological regressor zero-centered about the minimum and maximum values and the physiological regressor de-meaned. A regressor of no interest (BIO + SCRAM or HAP + ANG, as appropriate) was included to account for shared variance between trial types; it was convolved with a double-gamma HRF with temporal filtering applied and a temporal derivative added. All convolutions were applied prior to forming the interaction term; thereafter no further convolution was applied. Second-level fixed-effects analyses were used create within-subject estimates of the PPI term for OT versus PLC scans.

#### Group-level (mixed effects) analysis

Individual-specific OT > PLC contrasts were carried up into the group-level analysis. Group-level analysis was carried out at the whole-brain level using FLAME^77^,^78^ stages 1 and 2 with automatic outlier detection and deweighting^77^. Z (Gaussianized T/F) statistic images were thresholded using clusters determined by Z > 2.3 and a (corrected) cluster significance threshold of *p* = 0.05. Age (de-meaned), full scale IQ (de-meaned), and sex (dummy coded, female = 1) were included as covariates. In PPI analyses, the group mean estimate of the PPI regressor was assessed; for main effects analyses, the group mean estimate of the experimental contrast of interest was assessed. In additional exploratory individual differences analyses, the ADI-R social subscale score (de-meaned) and SRS Total T-score (de-meaned) were included in these models and evaluated on a whole-brain basis.

## Additional Information

**How to cite this article**: Gordon, I. *et al.* Intranasal Oxytocin Enhances Connectivity in the Neural Circuitry Supporting Social Motivation and Social Perception in Children with Autism. *Sci. Rep.* **6**, 35054; doi: 10.1038/srep35054 (2016).

## Supplementary Material

Supplementary Information

## Figures and Tables

**Figure 1:**
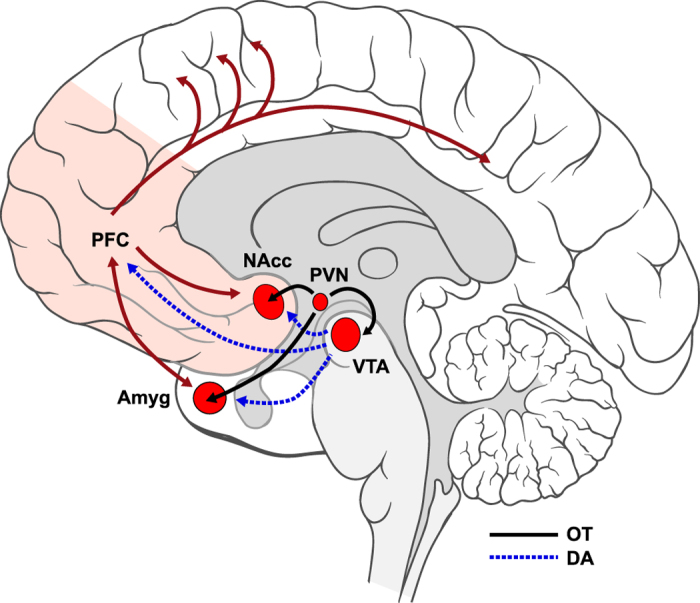
Schematic representation of oxytocinergic (OT; solid black) and mesocorticolimbic dopaminergic (DA; dashed blue) pathways. DA is synthesized in ventral tegmental area (VTA) and projects to nucleus accumbens (NAcc) and amygdala (Amyg) as well as prefrontal cortex (PFC). OT, which is synthesized in paraventricular nucleus (PVN), projects to key limbic sites on the DA pathway (VTA, NAcc, and Amyg), thus potentially allowing it to modulate DA activity. We note that while only neurotransmitter effects of OT are illustrated here, neuromodulatory effects via OT diffusion through extracellular space may also play a role in these processes. Via PFC, which is densely interconnected with many other regions of the brain (dark red solid lines), motivational factors may be able to influence attention and perception (see for review^79^). Brain art adapted from illustrations created by Patrick Lynch, medical illustrator, and C. Carl Jaffe, MD, cardiologist (available at https://commons.wikimedia.org/wiki/File:Brain_human_lateral_view.svg and https://commons.wikimedia.org/wiki/File:Brain_human_sagittal_section.svg) and licensed under a Creative Commons Attribution 2.5 Generic License, 2006 (CC BY 2.5).

**Table 1:** Demographic and phenotypic characteristics of the full sample and of subsamples analyzed after exclusion of motion-contaminated data.

	Full Sample	Biological Motion	Affective Voices
	*N* = 20	*n* = 14	*n* = 16
Age *M (SD)*	13.16 (2.79)	14.31 (2.26)	13.67 (2.76)
Full Scale IQ *M (SD)*	109.80 (18.17)	114.64 (18.24)	113.31 (17.4)
ADOS CSS *M (SD)*	7.20 (2.28)	6.86 (2.41)	7.06 (2.38)
ADI-R: A *M (SD)*	18.75 (4.89)	17.93 (4.87)	18.31 (5.15)
ADI-R: B *M (SD)*	14.70 (4.07)	14.00 (3.78)	14.69 (4.00)
ADI-R: C *M (SD)*	5.00 (2.36)	4.71 (2.43)	4.69 (2.27)
SRS total *t*-score *M (SD*)	77.11 (14.93)	73.14 (13.83)	73.50 (13.32)
Male *n*	17	11	13
Right-handed *n*	19	13	15
White *n*	16	12	13
Black *n*	2	1	1
More than one race *n*	2	1	2
On medication *n*	12	10	11
On psychiatric medication *n*	11	9	10

*Note*. Age is given in years. *ADOS CSS*: Autism Diagnostic Observation Schedule Calibrated Severity Score. *ADI-R*: Autism Diagnostic Interview-Revised. *ADI-R:A*: ADI-R Diagnostic algorithm A, “Qualitative Impairments in Reciprocal Social Behavior”. *ADI*-*R:B*: ADI-R Diagnostic algorithm B, “Qualitative Impairments in Communication and Language”. *ADI-R:C:* ADI-R Diagnostic algorithm C, “Restricted, Repetitive Behaviors and Interests”. *SRS*: Social Responsiveness Scale.

**Table 2:** Data quality summary statistics.

	Placebo		Oxytocin		Difference
	*M*	*SD*	*M*	*SD*	*p*
**Biological motion perception**					
Avg. absolute RMS movement (mm)	0.16	−0.07	0.27	−0.21	0.098
Avg. relative RMS movement (mm)	0.09	−0.04	0.09	−0.04	0.578
Motion outliers (% of total volumes)	5.10	−2.88	5.57	−3.73	0.599
Signal to fluctuation noise ratio	114.65	−18.78	107.99	−27.64	0.364
**Vocal affect perception**					
Avg. absolute RMS movement (mm)	0.30	−0.16	0.34	−0.24	0.393
Avg. relative RMS movement (mm)	0.14	−0.08	0.12	−0.08	0.300
Motion outliers (% of total volumes)	5.53	−4.25	3.63	−3.08	0.040
Signal to fluctuation noise ratio	115.1	−19.43	115.36	−26.36	0.941

*Note*. T-tests were used to calculate differences in means between treatment conditions. *RMS:* Root. mean squared.

**Table 3:** Cluster peaks and local maxima from main effects analysis of the biological motion perception and affective voice perception tasks, indicating regions where experimental contrasts of interest were greater under OT than PLC, and showed positive activation under OT.

Site	Hem	x	y	z	Z	k
Biological motion: BIO > SCRAM						
Posterior STS	R	46	−76	12	3.23	68
Affective voices: ANG > HAP						
Hippocampus (cornu ammonis)	R	32	−14	−12	4.25	378
Amygdala (centromedial)	R	22	−10	−8	3.37	—
Amygdala (laterobasal)	R	22	−8	−16	3.27	—
Amygdala (superficial)	R	18	−8	−12	3.1	—
Brainstem	R	14	−26	−12	3.27	94
Affective voices: HAP > ANG						
*n.s.*						

*Note.* MNI coordinates reported. *Hem*: Hemisphere. *L*: Left. *R*: Right. *Z*: Z-statistic. *k*: Voxel extent. *STS*: Superior temporal sulcus.

**Table 4:** Cluster peaks from PPI analysis of the Biological Motion and Affective Voices tasks, indicating regions where BIO > SCRAM or HAP > ANG connectivity was greater under OT than PLC.

Seed	Site	Hem	x	y	z	Z	k
Biological motion: BIO > SCRAM							
R NAcc							
	Frontal pole	R	40	48	−18	3.49	319
	vlPFC	R	16	20	−22	3.29	—
	vmPFC	—	4	32	−28	3.25	—
Affective voices: HAP > ANG							
L NAcc							
	Precuneus	—	6	−52	62	3.52	2825
	Ant. supramarginal gyrus	L	−66	−26	34	3.29	563
	Cuneus	—	−2	−84	30	3.30	361
	Crus I	R	42	−52	−26	3.32	273
R NAcc							
	Sup. lateral occipital cortex	R	26	−68	56	3.26	903
	Middle frontal gyrus	R	30	6	62	3.24	432
	Planum temporale	L	−62	−34	18	3.19	372
	Intracalcarine cortex	R	10	−88	2	3.28	338
	Angular gyrus	R	54	−48	30	3.45	337
L Amyg							
	Occipital pole	—	−8	−92	8	3.28	704
R Amyg							
	Precuneus	—	6	−60	12	3.33	1015

*Note*. MNI coordinates reported. *Hem*: Hemisphere. *L*: Left. *R*: Right. *Z*: Z-statistic. *k*: Voxel extent. *Ant*: Anterior. *Sup*: Superior. *Pos:* Posterior. *Amyg*: Amygdala. *NAcc*: Nucleus accumbens. *vlPFC:* ventrolateral prefrontal cortex. *vmPFC:* ventromedial prefrontal cortex.
