# Supplementary material for: Intranasal Oxytocin Enhances Connectivity in the Neural Circuitry Supporting Social Motivation and Social Perception in Children with Autism
Source: Sci Rep. 2016 Nov 15;6:35054. doi: 10.1038/srep35054 (PMC5109935; doi:10.1038/srep35054)

Supplementary Material

Intranasal Oxytocin Enhances Connectivity in the Neural Circuitry Supporting Social Motivation and Social Perception in Children with Autism

Ilanit Gordon[1,2]*†, Allison Jack[3]*†, Charlotte M. Pretzsch[1], Brent Vander Wyk[1], James F. Leckman[1], Ruth Feldman[1,2], & Kevin A. Pelphrey[3]

†Authors contributed equally to this work.

[1]Yale University

[2]Bar-Ilan University

[3]George Washington University

*Correspondence to: Dr. Ilanit Gordon, ilush.gordon@gmail.com, Ramat-Gan, Bar Ilan University, and to Dr. Allison Jack, allisonjack@gwu.edu, Washington, D.C., George Washington University.

Supplementary Results

Biological motion. Whole brain analyses were conducted to identify regions displaying a preferential response to coherent (BIO) versus scrambled (SCRAM) biological motion under either oxytocin (OT) or placebo (PLC) alone. Z-statistic images were thresholded using clusters determined by $Z > 2.3$ and a (corrected) cluster significance threshold of $p = 0.05$.

In examining BIO > SCRAM response under OT alone, we identified significant clusters of activation with peaks in cuneus, middle temporal gyrus, supplementary motor cortex, and supramarginal gyrus. See Table S3 and Fig. S1A. For BIO > SCRAM response under PLC alone, we identified significant clusters of activation with peaks in right supramarginal gyrus, brainstem, left supramarginal gyrus, left occipital pole, and left lateral occipital cortex. See Table S3 and Fig. S1B.

Affective voices. Whole brain analyses were conducted to identify regions displaying a preferential response to happy (HAP) versus angry (ANG) nonword vocalizations under either OT or PLC alone. Z-statistic images were thresholded using clusters determined by $Z > 2.3$ and a (corrected) cluster significance threshold of $p = 0.05$.

HAP > ANG. Under oxytocin alone, for the HAP > ANG contrast, we identified clusters of activation with peaks in right planum temporale and left Heschl's Gyrus. See Table S4 and Fig. S2A. Under PLC alone, we identified a significant cluster in right lateral occipital cortex. See Table S4 and Fig. S2B.

ANG > HAP. Under oxytocin alone, for the ANG > HAP contrast, we identified clusters of activation with peaks in the left callosal body, right precuneus, and right hippocampus. See Table S4 and Fig. S3. No significant results emerged for the ANG > HAP contrast under PLC.

Supplementary Tables

Table S1. Summary of functional imaging studies in oxytocin published between 2013-2016. A literature search using the Medline, EMBASE, and PsychINFO databases was performed. The following inclusion criteria were used: humans; English language and publication date from January 1, 2013 until April 15, 2016. The search terms used were “exp functional magnetic resonance imaging” AND “oxytocin/ih, na [Inhalational Drug Administration, Intranasal Drug Administration]”. The explode (exp) function allowed us to retrieve results containing subject headings together with narrower and more specific subheadings. For “functional magnetic resonance imaging”, these included chemical shift imaging; chemical shift imagings; functional mri; functional mris; functional magnetic resonance imaging; imaging, chemical shift; imaging, magnetic resonance; imaging, nmr; imagings, chemical shift; mr tomography; mri, functional; mri scan; mri scans; mris, functional; magnetic resonance imaging, functional; magnetic resonance imaging; magnetization transfer contrast imaging; nmr imaging; nmr tomography; proton spin tomography; scan, mri; scans, mri; shift imaging, chemical; shift imagings, chemical; tomography, mr; tomography, nmr; tomography, proton spin; zeugmatography; fmri. All extracted abstracts were reviewed for relevance to the question. Review articles, opinion pieces, and perspective papers were excluded.

Reference	Design	Paradigm/stimulus	Dose	Timing	Subjects*	Main Findings
Andari et al. (2016)	BS	Interactive ball game, Face-matching task	24 IU	30-45 min	20 (adults with ASD)	↑Occ Lobe (faces vs shapes), mOFC (fair); ↓INS (unfair), AMYG/HPPC (based on social context)
Ebner et al. (2016)	BS	Resting state	24 IU	70-90 min	79	↑FC: AMYG –mPFC (Oxy>Plc, M>F)
Eckstein et al. (2016)	BS	Pavlovian fear conditioning	24IU	30 min	97	↑CC subregions
Frijling et al. (2016)	BS, WS	Neutral/trauma-script driven imagery	40 IU	55-80 min	37 (adults; 11 days post-trauma)	↑FC: AMYG-INS; ↓FC: AMYG-vmPFC (regardless of imagery)
Nawijn et al. (2016)	WS	Monetary incentive delay (MID) task	40 IU	50-75 min	72 (adults with PTSD and trauma-exposed adults without PTSD)	↑STR, ACC, INS (to reward/loss anticip.)
Aoki et al. (2015)	WS, CO	Facial (non)verbal socio-emotional paradigm	24 IU	40 min	40 (male adults with HF ASD)	↑vmPFC (rel. to NAA levels)
Eckstein et al. (2015)	BS	Pavlovian fear conditioning	24 IU	30 min	62	↑PFC (cond. fear in early extinct. phase), ↓AMYG (across phases)
Feng et al. (2015)	BS	Iterated Prisoner’s Dilemma game	24 IU	Not reported	304	↑CPU (male), ↓CPU (female)
Feng et al. (2015)	BS	Prisoner’s Dilemma task	24 IU	Not reported	204	↑vCN (reciprocated coop. in men), ↓vCN (reciproc. coop. in women); effects specific to rs53576 GG genotype

Feng et al. (2015)	BS	Prisoner's Dilemma game	24 IU	Not reported	153 (men)	Intranasal Oxy did not interact with neuroticism to influence neural response to pos./neg. social interactions
Kanat et al. (2015)	BS	Emotional/neutral eyes and faces	24 IU	55 min	50 (men)	↓AMYG (masked fearful eye whites)
Kovács et al. (2015)	BS	Resting state	~35 IU 4.6 times/week for ~9 weeks (off-label use)	N/A	82 (41 Oxy users and 41 matched controls)	↑FC: AMYG-ACC
Kumar et al. (2015)	CO	Resting state	24 IU	45 min	15 (men)	↓Degr. central.: PREC, ↓FC: bilat. AMYG, PREC-bilat. AMYG
Shin et al. (2015)	CO	Facial emotion-recognition paradigm	40 IU	45 min	32 (16 adult males with schizophrenia and 16 controls)	↓AMYG (emotional faces) in SCZ, ↑AMYG (emotional faces) in controls
Watanabe et al. (2015)	CO	Social-judgement task and resting state	48 IU/day (2 doses/day) for 6 weeks	40 min	20 (men with HF ASD)	↑FC: ACC-dmPFC, ↑ACC, dmPFC
Zunhammer et al. (2015)	CO	Noxious experimental heat	32 IU	40 min	30 (men)	↓AMYG (across temp)
Aoki et al (2014)	CO, WS	Modified Sally-Anne task	24 IU	40 min	20 (men with ASD)	↑aINS (inferring others' social emotions)
Dodhia et al. (2014)	CO	Resting state	24 IU	45 min	36 (18 adults with GSAD, 18 controls)	↑FC: bilat. AMYG-ACC/mPFC (in GSAD vs. controls)
Eckstein et al. (2014)	BS	Montreal Imaging Stress Task	24 IU	30 min	60 (men)	↑PREC, CC
Riem et al. (2014)	BS, WS	Adapted RMET	16 IU	60 min	50 (women)	↑INS, STG (in indiv. with harsh caregiving experiences)
Rilling et al. (2014)	BS	Prisoner's dilemma task	24 IU	43 (40-50) min	87 (women)	↓AMYG (recipr. human coop.)
Voorthuis et al. (2014)	BS	Adapted version of Infant Facial Expression of Emotions from Looking (IFEEL) at Pictures	16 IU	50-60 min	50 (women)	↑IFG, MTG, STG
Watanabe et al. (2014)	WS, CO	Social psychological task	24 IU	40 min	40 (men with HF ASD)	↑mPFC
Watanabe et al. (2014)	WS, CO	Facial (non)verbal socio-emotional paradigm	24 IU	40 min	40 (men with HF ASD)	↑mPFC

Domes et al. (2013)	WS, CO	Face-matching task, House-matching task	24 IU	45 min	28 (14 Asperger, 14 controls)	↑AMYG (for faces in AS)
Gordon et al. (2013)	CO	Modified RMET	12/18/24 IU (age dependent)	45 min	17 (children adolescents with ASD)	↑STR, MFG, SFG, mPFC, OFC, STS; PG, STR, NAcc, Cll, Pons, pCC/PREC, PHC, IPL, STS (↑ for social, ↓ for non-social)
Groppe et al. (2013)	BS	Social incentive task	~26 IU	30 min	28 (women)	↑VTA (friendly face= social reward, angry face= social punishment)
Scheele et al. (2013)	WS	Faces of long-term romantic partners	24 IU	30 min	20 (men)	↑VTA, NAcc (partner vs. unfamiliar), ↑lNAcc (partner vs. familiar women)

Note. *Unless specified otherwise, subjects are healthy adults (men and women). *IU*: International Units in total. *min*: minutes. *BS*: Between-subject. *WS*: Within-subject. *CO*: Cross-over. *ASD*: Autism Spectrum Disorder. *Occ*: occipital. *mOFC*: medial orbitofrontal cortex. *INS*: Insula. *AMYG*: amygdala. *HPPC*: hippocampus. *mPFC*: medial prefrontal cortex. *Oxy*: Oxytocin. *Plc*: Placebo. *M*: Male. *F*: Female. *CC*: cingulate cortex. *FC*: functional connectivity. *PTSD*: post-traumatic stress disorder. *STR*: striatum. *ACC*: anterior cingulate cortex. *HF ASD*: high-functioning autism. *vmPFC*: ventromedial prefrontal cortex. *NAA*: N-Acetyl-Aspartate. *PFC*: prefrontal cortex. *CPU*: Caudate/putamen. *vCN*: ventral caudate nucleus. *PREC*: precuneus. *bilat*: bilateral. *aINS*: anterior INS. *GSAD*: Generalised Social Anxiety Disorder. *STG*: superior temporal gyrus. *IFG*: inferior frontal gyrus. *MTG*: middle temporal gyrus. *RMET*: Reading the Mind in the Eyes Task. *pCC*: posterior cingulate cortex. *PHC*: parahippocampus. *IPL*: inferior parietal lobe. *STS*: superior temporal sulcus. *VTA*: ventral tegmental area. *NAcc*: Nucleus Accumbens.

Table S2. Cluster peaks of regions from main effects analysis of the biological motion perception and affective voice perception tasks, indicating regions where the experimental condition of interest showed a significantly attenuated deactivation under OT than PLC.

Site	Hem	x	y	z	Z	k
Biological motion: BIO > SCRAM						
Supracalcarine cortex	L	-2	-84	10	3.45	752
Affective voices: ANG > HAP						
Precuneus	R	10	-54	24	4.01	1231
Fornix	R	28	-34	4	3.65	
Callosal body	R	24	-38	26	3.59	
Optic radiation	R	28	-72	16	3.65	711
Superior parietal lobule	R	30	-66	58	3.48	

Note. MNI coordinates reported. *Hem:* hemisphere. *Z*: z-statistic. *k*: voxel extent.

Table S3. Cluster peaks and local maxima of regions with significant response to the BIO > SCRAM contrast, under OT alone and under PLC alone.

Site	Hem	x	y	z	*Z*	*k*
BIO > SCRAM under OT						
Cuneal Cortex	--	-4	-82	20	4.33	1445
Supracalcarine Cortex	--	-2	-86	10	3.69	--
Intracalcarine Cortex	L	-14	-72	10	3.62	--
Middle Temporal Gyrus	L	-58	-58	8	4.68	815
Lateral Occipital Cortex	L	-60	-68	6	4.54	--
Supplementary Motor Cortex	R	10	-14	46	3.8	768
Postcentral Gyrus	R	24	-36	56	3.73	--
Cingulate Gyrus	L	-12	-18	38	3.67	--
Precentral Gyrus	--	4	-30	54	3.64	--
Supramarginal Gyrus	R	64	-34	30	4.65	573
BIO > SCRAM under PLC						
Supramarginal Gyrus	R	54	-38	16	4	848
Inferior parietal lobule	R	68	-34	26	3.81	--
Parietal Operculum Cortex	R	50	-32	30	3.79	--
Brainstem	R	8	-34	-2	3.91	752
Amygdala	R	26	-2	-18	3.72	--
Thalamus	R	12	-36	0	3.56	--
Supramarginal Gyrus	L	-58	-32	50	3.91	597
Occipital Pole	R	36	-94	0	4.35	443
Occipital Pole	L	-28	-100	-2	3.82	359
Lateral Occipital Cortex	L	-38	-90	-10	3.63	--
Lateral Occipital Cortex	L	-56	-64	4	3.72	320

Note. MNI coordinates reported. *Hem*: Hemisphere. *L*: Left. *R*: Right.

Table S4. Cluster peaks and local maxima of regions with significant response to either the HAP > ANG or ANG > HAP contrast, under OT alone and under PLC alone.

Site	Hem	x	y	z	*Z*	*k*
HAP > ANG under OT						
Planum Temporale	R	58	-20	12	4.29	1486
Superior Temporal Gyrus	R	64	-16	2	4.2	--
Heschl's Gyrus	L	-44	-28	12	4.3	722
Planum Temporale	L	-64	-28	14	3.91	--
Parietal Operculum Cortex	L	-48	-30	14	3.9	--
Superior Temporal Gyrus	L	-68	-22	8	3.71	--
HAP > ANG under PLC						
Lateral occipital cortex	R	28	-68	56	3.84	895
Superior parietal lobule	R	24	-50	56	3.6	--
ANG > HAP under OT						
Callosal body	L	-28	-56	12	3.68	937
Optic radiation	L	-28	-66	6	3.55	--
Temporal occipital fusiform	L	-34	-52	-8	3.43	--
Precuneus	R	10	-52	22	3.48	662
Callosal body	R	22	-46	10	3.43	--
Premotor cortex	R	32	-16	54	3.37	--
Hippocampus	R	28	-18	-12	3.63	487
Amygdala	R	22	-4	-20	3.54	--
ANG > HAP under PLC						
n.s.						

Note. MNI coordinates reported. *Hem*: Hemisphere. *L*: Left. *R*: Right. *n.s.*: Non-significant

Table S5. Cluster peaks and local maxima of regions that demonstrated significantly greater activation under OT than PLC for both HAP > fixation and ANG > fixation.

Site	Hem	x	y	z	Z	k
Planum temporale	L	-44	-32	8	3.80	1287
Superior temporal gyrus	L	-64	-20	4	3.67	--
Superior temporal gyrus	R	46	-30	4	4.00	1055
Supramarginal gyrus	R	58	-40	16	3.88	--
Heschl's gyrus	R	56	-14	8	3.79	--

Note. MNI coordinates reported. *Hem:* hemisphere. *Z*: z-statistic. *k*: voxel extent.

Table S6. Local maxima resulting from PPI analysis of the affective voices task, indicating regions where HAP > ANG connectivity was greater under OT than PLC.

Seed	Site	Hem	x	y	z	Z	k
Left NAcc							
	Precuneus	--	6	-52	62	3.52	2825
	Sup. lateral occipital cortex	R	14	-66	54	3.36	--
	Sup. parietal lobule	L	-26	-58	62	3.31	--
	Postcentral gyrus	L	-28	-36	64	3.29	--
	Ant. supramarginal gyrus	L	-66	-26	34	3.29	563
	Heschl's gyrus	L	-50	-18	8	3.27	--
	Planum temporale	L	-52	-30	10	3.19	--
	Parietal operculum	L	-62	-28	18	3.19	--
	Postcentral gyrus	L	-60	-22	36	3.17	--
	Cuneus	--	-2	-84	30	3.30	361
	Occipital pole	R	16	-94	26	3.29	--
	Crus I	R	42	-52	-26	3.32	273
	Temporo-occipital fusiform	R	26	-48	-18	3.29	--
Right NAcc							
	Sup. lateral occipital cortex	R	26	-68	56	3.26	903
	Pos. supramarginal gyrus	R	50	-42	60	3.21	--
	Precuneus	--	4	-70	50	3.10	--
	Middle frontal gyrus	R	30	6	62	3.24	432
	Sup. frontal gyrus	R	26	22	58	3.20	--
	Planum temporale	L	-62	-34	18	3.19	372
	Ant. supramarginal gyrus	L	-62	-28	20	3.16	--
	Parietal operculum	L	-58	-32	18	3.11	--
	Postcentral gyrus	L	-62	-24	42	3.08	--
	Intracalcarine cortex	R	10	-88	2	3.28	338
	Lingual gyrus	R	12	-86	-2	3.25	--
	Occipital pole	--	4	-90	0	3.21	--
	Angular gyrus	R	54	-48	30	3.45	337
Left Amyg							
	Occipital Pole	--	-8	-92	8	3.28	704
	Supracalcarine Cortex	--	4	-86	8	3.23	--
	Intracalcarine Cortex	R	10	-82	6	3.17	--
Right Amyg							
	Precuneus	--	6	-60	12	3.33	1015
	Occipital Pole	R	12	-98	4	3.25	--
	Supracalcarine Cortex	--	2	-80	10	3.21	--

Note. MNI coordinates are reported. Hem: Hemisphere. L: Left. R: Right. Z: Z-statistic. k: Voxel extent. Ant: Anterior. Sup: Superior. Post: Posterior. Amyg: Amygdala. NAcc: Nucleus accumbens.

Table S7. Regions where HAP > ANG PPI values were associated with social symptom severity as measured by SRS total *t* score.

Seed	Site	Hem	x	y	z	Z	k
Positive associations							
Left NAcc							
	Ant. Temporal Fusiform Cortex	R	36	-6	-40	3.54	732
	Temporal Pole	L	-42	14	-26	3.34	651
R NAcc							
	Ant. Inferior Temporal Gyrus	R	40	-2	-38	3.48	985
	Ant. Temporal Fusiform Cortex	L	-30	-2	-36	3.36	386
	Frontal Medial Cortex	L	-10	36	-22	3.22	287
R Amyg							
	Inferior Lateral Occipital Cortex	L	-46	-76	-12	3.92	341
	Frontal Medial Cortex	--	0	36	-22	3.36	312
Negative associations							
n.s.							

Note. MNI coordinates are reported. *Hem*: Hemisphere. *L*: Left. *R*: Right. *Z*: Z-statistic. *k*: Voxel extent. *Ant*: Anterior. *Amyg*: Amygdala. *NAcc*: Nucleus accumbens.

Table S8. Regions where HAP > ANG PPI values were associated with social symptom severity as measured by ADI-R diagnostic algorithm A, “Qualitative Abnormalities in Reciprocal Social Interaction.”

Seed	Site	Hem	x	y	z	Z	k
Positive associations							
Left NAcc							
	Frontal Pole	R	24	42	38	3.92	279
R NAcc							
	Frontal Pole	R	24	44	38	3.8	491
Negative associations							
Left NAcc							
	vmPFC	R	8	26	-16	3.69	722
	Ant. Parahippocampal Gyrus	L	-28	-16	-32	3.43	379
Left Amyg							
	vmPFC	L	-2	36	-12	3.48	1294

Note. MNI coordinates are reported. *Hem*: Hemisphere. *L*: Left. *R*: Right. *Z*: Z-statistic. *k*: Voxel extent. *Ant*: Anterior. *Amyg*: Amygdala. *NAcc*: Nucleus accumbens. *vmPFC*: ventromedial prefrontal cortex.

Table S9. Detailed summary of the types of medication used by participants in the full sample and the subsamples analyzed for the biological motion perception and affective voice perception tasks.

	Full Sample	Biological Motion	Affective Voices
	N = 20	n = 14	n = 16
Alpha agonist	2	2	2
Anticonvulsant/mood stabilizer	1	1	1
Anti-inflammatory	2	2	2
Antihistamine	2	1	1
Antipsychotic/anti-irritability	5	4	4
Growth hormone	1	1	0
Inhaled corticosteroid	1	0	1
SNRI	1	1	1
SSRI	3	3	3
Stimulant	3	2	3

Note. *Alpha agonists:* guanfacine, clonidine. *Anticonvulsant/mood stabilizer:* lamotrigine. *Anti-inflammatory:* montelukast. *Antihistamines:* fexofenadine, cetirizine. *Antipsychotic/anti-irritability drugs:* aripiprazole, risperidone. *Growth hormone:* somatropin. *Inhaled corticosteroid:* mometasone. *SNRI (serotonin-norepinephrine reuptake inhibitor):* venlafaxine. *SSRIs (selective serotonin reuptake inhibitors):* fluoxetine, sertraline. *Stimulants:* amphetamine/dextroamphetamine, dexmethylphenidate, lisdexamfetamine.

Figure S1. Brain regions in which activation was significantly higher while viewing coherent (BIO) versus scrambled (SCRAM) biological motion, under either **A)** oxytocin (OXT) only, or **B)** placebo (PLC) only. *Z*-statistic maps are overlaid on the MNI standard template in radiological orientation, with every other axial slice depicted from z = 0 to z = 94.

A. BIO > SCRAM: OXT

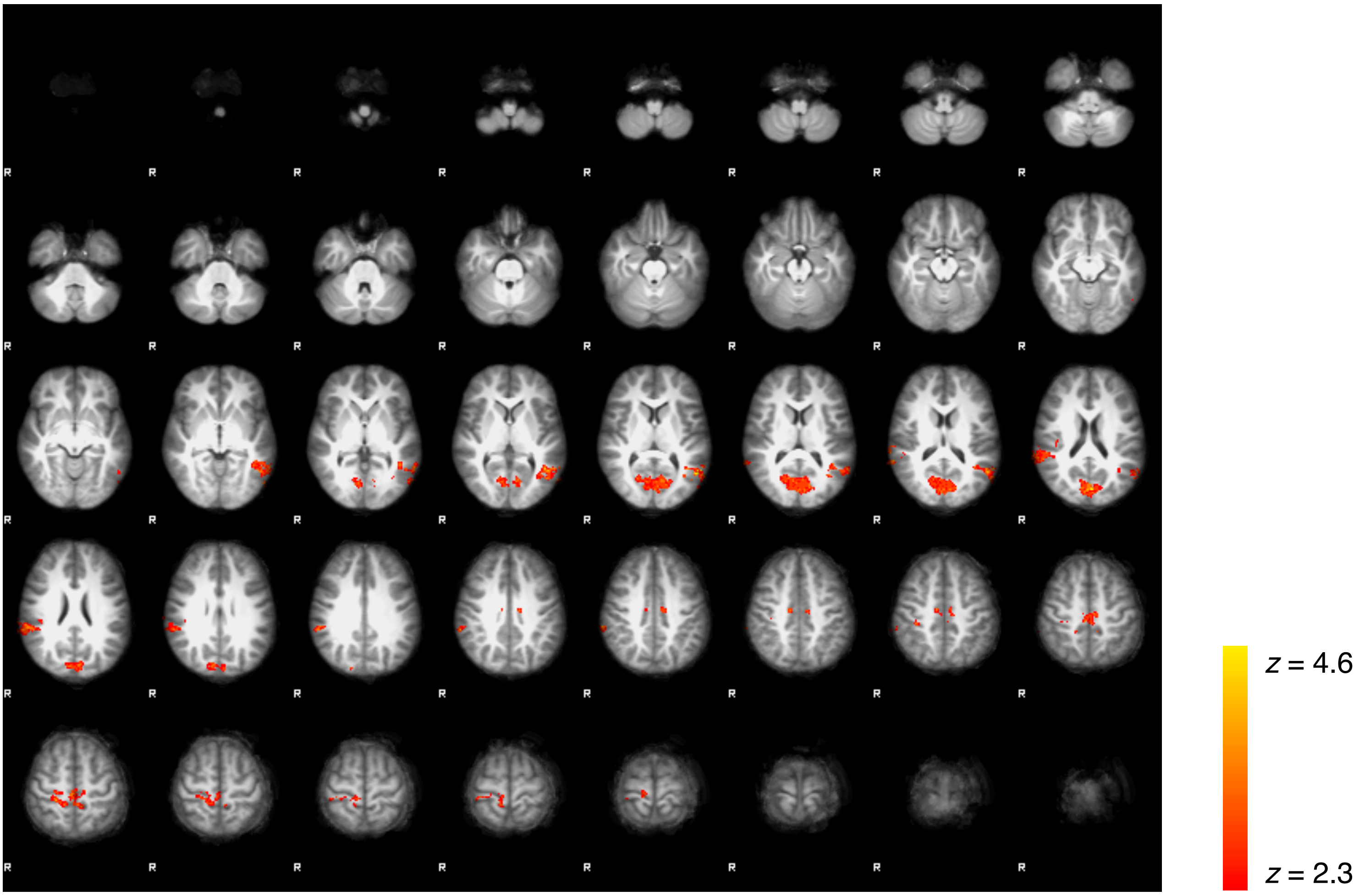

B. BIO > SCRAM: PLC

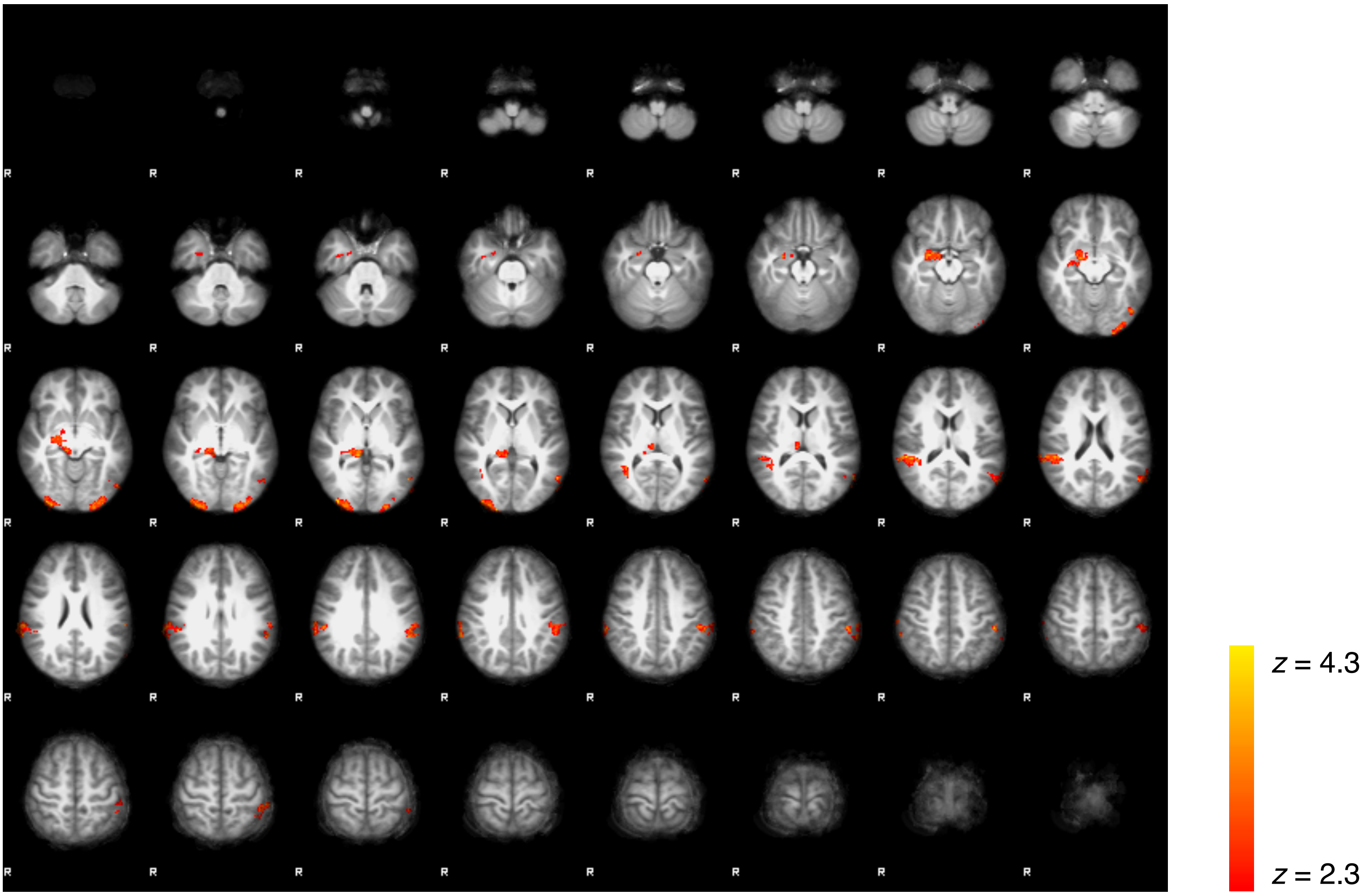

Figure S2. Brain regions in which activation was significantly higher while listening to happy (HAP) versus angry (ANG) vocal stimuli, under either **A)** oxytocin (OXT) only, or **B)** placebo (PLC) only. *Z*-statistic maps are overlaid on the MNI standard template in radiological orientation, with every other axial slice depicted from z = 0 to z = 78.

A. HAP > ANG: OXT

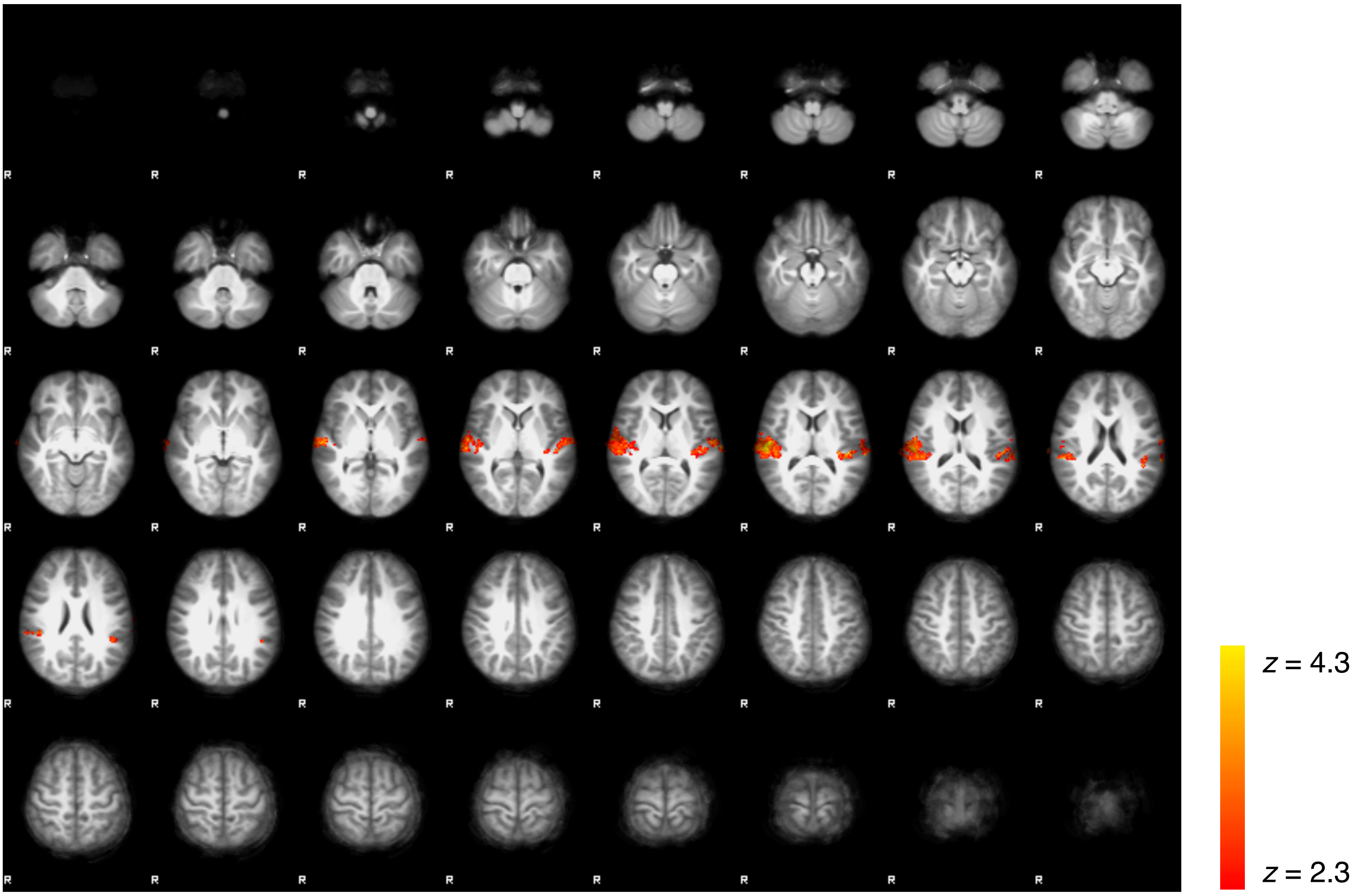

B. HAP > ANG: PLC

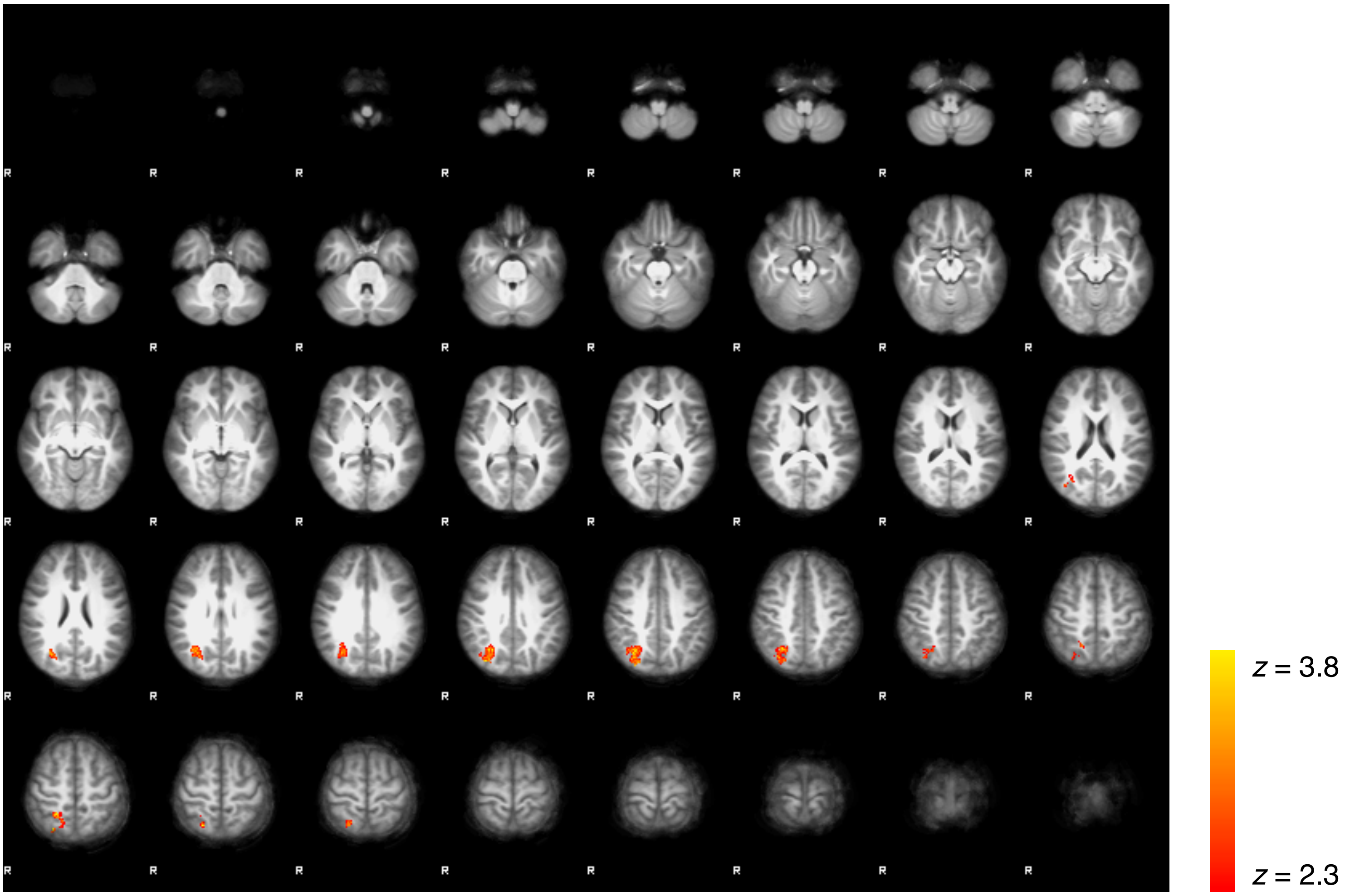

Figure S3. Brain regions in which activation was significantly higher while listening to angry (ANG) versus happy (HAP) vocal stimuli, under oxytocin (OXT) only. *Z*-statistic maps are overlaid on the MNI standard template in radiological orientation, with every other axial slice depicted from z = 0 to z = 78.

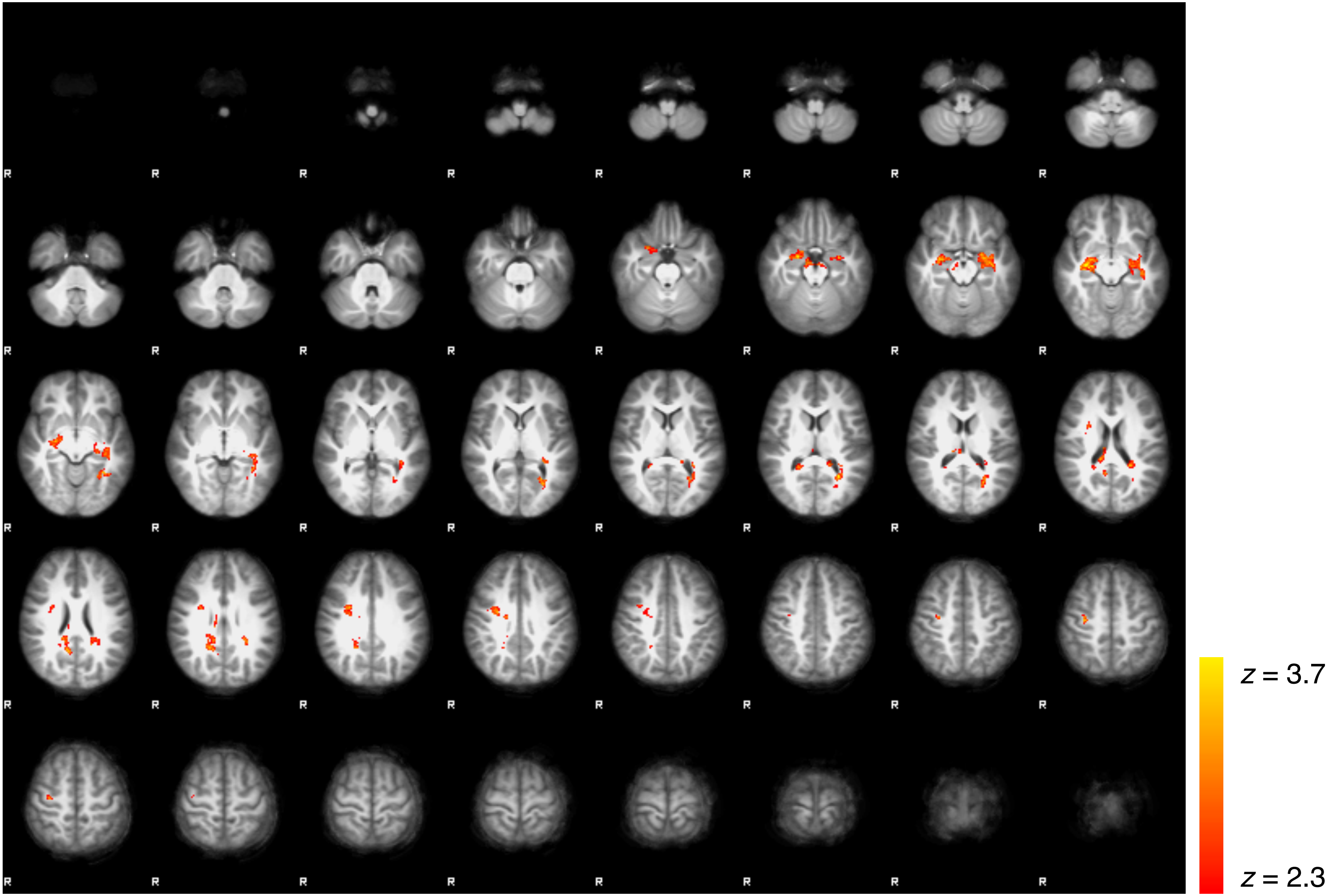

Figure S4. Results of conjunction analysis, indicating regions with significantly greater response to both ANG > fixation and HAP > fixation under oxytocin than placebo. *Z*-statistic maps are overlaid on the MNI template in radiological orientation.

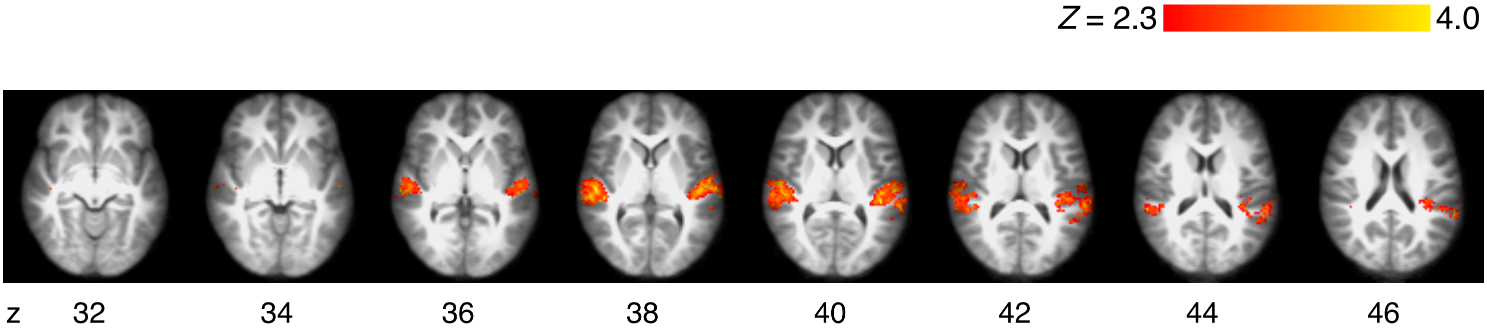

Supplement: Supplementary Information [file srep35054-s1.pdf]
